# Conversion of Aryl Iodides into Aryliodine(III) Dichlorides by an Oxidative Halogenation Strategy Using 30% Aqueous Hydrogen Peroxide in Fluorinated Alcohol

**DOI:** 10.3390/molecules15042857

**Published:** 2010-04-20

**Authors:** Ajda Podgoršek, Jernej Iskra

**Affiliations:** Department of Physical and Organic Chemistry, “Jožef Stefan” Institute, Jamova cesta 39, 1000 Ljubljana, Slovenia; E-Mail: ajda.podgorsek@ijs.si (A.P.)

**Keywords:** oxidative halogenation, hydrogen peroxide, fluorinated alcohol, hypervalent iodine halides, chlorination

## Abstract

Oxidative chlorination with HCl/H_2_O_2_ in 1,1,1-trifluoroethanol was used to transform aryl iodides into aryliodine(III) dihalides. In this instance 1,1,1‑trifluoroethanol is not only the reaction medium, but is also an activator of hydrogen peroxide for the oxidation of hydrochloric acid to molecular chlorine. Aryliodine(III) dichlorides were formed in 72–91% isolated yields in the reaction of aryl iodides with 30% aqueous hydrogen peroxide and hydrochloric acid at ambient temperature. A study of the effect that substituents on the aromatic ring have on the formation and stability of aryliodine(III) dichlorides shows that the transformation is easier to achieve in the presence of the electron-donating groups (*i.e.* methoxy), but in this case the products rapidly decompose under the reported reaction conditions to form chlorinated arenes. The results suggest that oxidation of hydrogen chloride with hydrogen peroxide is the initial reaction step, while direct oxidation of aryl iodide with hydrogen peroxide is less likely to occur.

## 1. Introduction

With the development of the chemistry of hypervalent iodine compounds, aryliodine(III) dihalides, like (dichloroiodo)benzene, have received growing interest in organic synthesis as mild and selective chlorinating and oxidizing agents. Due to the fact that they are readily available and can be handled safely, such compounds offer a practical advantage over gaseous chlorine. Moreover, they serve as starting substrates for the synthesis of other hypervalent iodine compounds, *i.e.*, iodosylarenes, (diacyloxyiodo) arenes, (difluoroiodo) arenes and iodylarenes, which are themselves important synthetic reagents [1,2,3,4]. However, the broader application of (dichloroiodo) benzene, as well as other hypervalent iodine compounds, has been limited due to the often tedious separation of the residual iodobenzene from the desired products. To circumvent this problem, several recyclable aryliodine (III) dichlorides have been developed for solid-liquid or liquid-liquid organic/aqueous phase separation [5,6,7,8]. Recently, we have reported a synthesis and characterization of several fluorous aryl and alkyl iodine (III) dichlorides and detailed their use as recyclable chlorinating reagents for representative types of organic molecules [9].

The most frequently encountered representative amongst the (dichloroiodo) arenes is (dichloroiodo) benzene, that was first prepared by Willgerodt [10] in 1886 by passing chlorine through a solution of iodobenzene in an organic solvent, usually either dichloromethane or chloroform at a low temperature; this remains the most general method [1,9,11,12,13]. However, the inconvenient use of hazardous, toxic and corrosive gaseous chlorine can be avoided by using different chlorinating agents under various reaction conditions [1,2,13,14]. For instance, oxidative halogenation has emerged as an environmentally more benign process *via* the *in situ* formation of molecular halogen from the oxidation of an halide with a suitable oxidant [15]. Therefore, mono and biphasic oxidative procedures based on generating chlorine from concentrated hydrochloric acid used either as a separate phase or co-solvent in the presence of an oxidant have been developed [1,2,3,4,14]. Skulski and others have used strong oxidants, such as KMnO_4_, activated MnO_2_ [16], KClO_3_ [17], concentrated HNO_3_ [16], Na_2_S_2_O_8_ [18], CrO_3_ [19], NaClO_2_ and NaClO [20], usually in excess, to synthesize various (dichloroiodo) arenes. Oxidative methods for the synthesis of (dichloroiodo) arenes have also been extended to derivatives of hydrogen peroxide as safer, easier to handle and environmentally less harmful oxidants, like sodium perborate (NaBO_3_·H_2_O or NaBO_3_·4H_2_O, SPB) [16,21] sodium percarbonate (Na_2_CO_3_·1.5H_2_O_2_, SPC) [16] and urea hydrogen peroxide (NH_2_CONH_2_·H_2_O_2_, UHP) [16,22]. With an 400% of excess of NaBO_3_·4H_2_O in CH_3_CN or CCl_4_ various (dichloroiodo)arenes were prepared in 60–98% yields at room temperature [21], meanwhile a 200% excess of NaBO_3_·H_2_O was sufficient to obtain ArICl_2 _in similar yields (63–99%) in an AcOH-Ac_2_O medium [16]. Under solvent-free conditions using UHP at 85 °C and an excess of concentrated HCl, the preparation of ArICl_2_ in good to excellent yields was achieved [22]. All of the aforementioned methods use the iodoarenes as starting substrates and the reactions are generally performed in either AcOH or CCl_4_ at 0–5 °C, except in the case of Na_2_S_2_O_8_ where preheating to 45–50 °C is necessary. Alternatively, procedures for a one-pot conversion of arenes to (dichloroiodo)arenes were also reported using, in the first step, either a combination of I_2_/CrO_3_ [23], I_2_/NaIO_4_ or NaIO_3_ [24] in Ac_2_O/AcOH/conc. H_2_SO_4_ medium, followed by treatment with concentrated HCl.

However, several of these methods are effective only for selected iodoarenes and especially aryl iodine (III) dichlorides with electron-withdrawing substituents on the aromatic nucleus are usually obtained in lower yields. The main disadvantage of the current methods lies in the use of a very strong and environmentally harmful oxidant, usually in excess amounts, which additionally complicates the purification of the product. Furthermore, many oxidants have to be previously activated and added portion-wise to a vigorously stirred reaction mixture of iodoarene in an organic solvent (AcOH or chlorinated solvent) to control the exothermic reactions and to enable the slow reaction of the Cl_2_ generated *in situ* with the substrate.

In continuation of our research on sustainable and ecologically more acceptable halogenation protocols [25,26,27,28,29], we investigated the use of 30% aqueous hydrogen peroxide and hydrochloric acid for the synthesis of various aryliodine (III) dichlorides from the corresponding aryl iodides. The use of dilute aqueous H_2_O_2_ offers advantages over solid UHP since it is cheaper and since the only reaction byproduct is water, the atom economy of the process is higher. In 2003, Neumann *et al*. reported oxidative chlorination in a HCl-H_2_O_2_ system in 1,1,1-trifluoroethanol (CF_3_CH_2_OH, TFE), whose activating effect enabled the use of 1.5 equivalents of HCl for the quantitative chlorination of non-activated aromatics at room temperature [30]. With DFT calculations it was shown that TFE stabilizes the polar transition state by acting as a charge template with charges complementary to those in the transition state and therefore lowers its energetic barrier relative to EtOH by ~6 kcal/mol. Encouraged by these results, we decided to apply TFE as a template catalyst for the activation of H_2_O_2_ for the oxidative chlorination of iodoarenes at room temperature and for substitute activation by either heating or using an acetic acid medium.

## 2. Results and Discussion

### 2.1. Synthesis of Aryl Iodine (III) Dichlorides

Iodobenzene (**1a**) was chosen as the initial substrate to be transformed to the corresponding iodobenzene dichloride (**1b**) by a novel oxidative chlorination procedure (Table 1). Iodobenzene was suspended in 1,1,1-trifluoroethanol into which was then added 1.0 mol equiv. of 30% aqueous H_2_O_2_ and 2.0 mol equiv. of 37% aqueous hydrochloric acid (Entry 1, Method A). The heterogeneous mixture was then stirred at room temperature for 4 h. The solvent was removed under reduced pressure to give **1b** as a yellowish precipitate which was filtered off, washed with distilled water and air-dried to give pure **1b** in 80% yield. In the next step, the quantities of H_2_O_2_ and HCl were increased to 2.0 and 4.0 mol equiv., respectively (Method B) which enhanced the isolated yield of **1b** to 89%. The yields obtained in this oxidative procedure were then compared to the classical method, where a solution of **1a** in hexane was saturated with molecular Cl_2_ and stirred for 3 h at ambient temperature (Method D). In this case, (dichloroiodo) benzene (**1b**) was obtained in 96% yield. 

Next, the same procedure was applied to the synthesis of other aryl iodine (III) dichlorides **2b-8b** and the effect that the substituents on the aromatic ring have on the yield was studied. Application of Method A for chlorination of aryliodides **2a** and **3a** with either a methyl or *t*-butyl group at the *para* position gave lower yields of **2b** and **3b**, probably because of the lower solubility of **2a** and **3a** in TFE (Entries 2 and 3). To obtain yields of **3b** comparable to those obtained using molecular chlorine, an excess of H_2_O_2_ and HCl had to be used (Method C). A similar effect of lowering the yield of formation of aryl iodine(III) dichlorides in the presence of alkyl substituents on the aromatic ring was also observed using other oxidants, like CrO_3 _ [19] or Na_2_S_2_O_3_ [18] where **2b** was obtained with up to 14% lower yield as **1b**. On the contrary, reactions with SPB [21] or UHP [31] as oxidants gave **2b** in slightly higher yields (up to 5%) as **1b**. 4,4’-Bis(dichloroiodo)biphenyl (**4b**) was isolated in 89% yield in the reaction of **4a** with 4.0 mol equiv. of H_2_O_2 _and 4.0 equiv. of H_2_O_2_ (Entry 4).

**Scheme 1:**
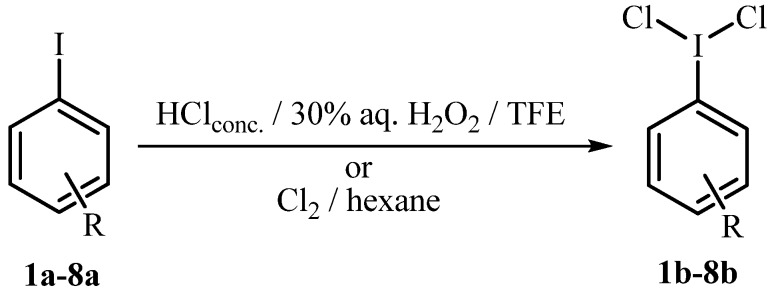
Synthesis of aryliodine(III) dichlorides **1b-8b** through oxidative chlorination or directly with Cl_2_.

**Table 1:** Synthesis of Aryliodine(III) Dichlorides **1b-8b**.

Entry	Substrate	Method ^a^	Time (h)	Yield ^b^ (%)
1	**1a**, R=H	A	4	**1b**, 80
		B	4	89
		D	3	96
2	**2a**, R=4-CH_3_	A	16	**2b**, 66
		B	16	90
		D	3	92
3	**3a**, R=4-C(CH_3_)_3_	A	19	**3b**, 55
		C	19	80
		D	19	85
4	**4a**, R=4-(4-I-C_6_H_4_)	B	5	**4b**, 85
		C	19	89
		D	10	96
5	**5a**, R=3-COOH	A	5	**5b**, 40
		B	20	78
		D	10	82
6	**6a**, R=3-NO_2_	B	14	**6b**, 20
		C	20	72
		D	19	76
7	**7a**, R=3-Cl	B	19	**7b**, 59
		C	20	85
		D	19	90
8	**8a**, R=4-Cl	B	14	**8b**, 50
		C	19	85
		D	19	92

Reaction conditions: Iodoarene **1a-8a** (1.0 mmol), ambient temperature. ^a^Method A: conc. HCl (2.0 mmol), 30% aq. H_2_O_2_ (1.0 mmol), TFE (1.0 mL); Method B: conc. HCl (4.0 mmol), 30% aq. H_2_O_2_ (2.0 mmol), TFE (1.0 mL); Method C: conc. HCl (4.0 mmol), 30% aq. H_2_O_2_ (4.0 mmol), TFE (1.0 mL). Method D: Cl_2_ (excess), hexane (10 mL). ^b^Isolated yield.

The presence of electron-withdrawing substituents on the aromatic nucleus led to a slower reaction and reduced yields of (dichloroiodo)arenes. In the case of 3-iodobenzoic acid (**5a**) 4.0 mol equiv. of HCl had to be used and a longer reaction time was necessary to obtain **5b** in 78% yield (Entry 5). Similarly, the reaction involving molecular chlorine was also less effective and only 82% of **5b** was isolated. With more powerful deactivating groups, like Cl and NO_2_, a further excess of reagents was required (Method C) to produce **6b** and **7b** in 72% and 85% yield, respectively (Entry 6 and 7). No significant difference in reactivity was observed with a chloro substituent at either the *meta* or *para* position (Entry 8). Iodoarenes **1a-8a** were also chlorinated with molecular chlorine in hexane (Method D), which gave slightly higher yields of (dichloroiodo)arenes **1b-8b**. However, the yields obtained in HCl/H_2_O_2_/TFE are comparable with those obtained by other oxidative methods involving stronger oxidants.

### 2.2. Chlorination of Activated Aryliodides with HCl/H_2_O_2_/TFE and with Molecular Chlorine

**Scheme 2:**
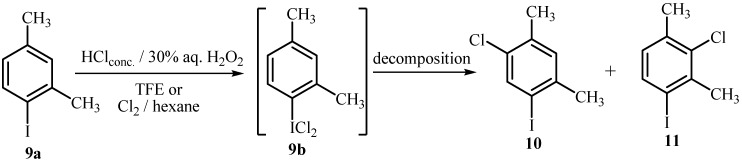
Chlorination of 2,4-dimethyliodobenzene (**9a**).

The chlorination of iodoarenes with electron-donating substituents was also studied since these substrates have an activated aromatic ring suitable for chlorination as well as an iodine atom for oxidation. For substrates having two or three methyl groups on the aromatic nucleus chlorination was performed using either the oxidative procedure or with molecular chlorine (Scheme 2). 

2,4-Dimethyl-iodobenzene (**9a**) was treated with 2.0 mol equiv. of HCl and 1.0 mol equiv. of H_2_O_2_ in TFE and the reaction was followed by NMR. After 0.5 h only the signals corresponding to **9b** were observed, which was formed in 17% yield and it increased to 46% after 2 h as shown by its characteristic signal in the ^1^H-NMR spectra at 8.07 ppm (d, *J* = 8 Hz) (Table 2, Entry 1 and 2). The same sample was left for 24 hours at ambient temperature. The analysis revealed that **9b** had started to decompose into a mixture of ring chlorinated products (Entry 3). The reaction mixture was isolated, whereupon ^1^H-NMR spectroscopy revealed the presence of 30% of 1-chloro-5-iodo-2,4-dimethylbenzene (**10**) and 37% of 2-chloro-4-iodo-1,3-dimethylbenzene (**11**). Chlorinated products were isolated by preparative TLC in a 35% yield as a mixture of **10** and **11**, the structures of which were determined by comparison of the ^1^H-NMR shifts with the literature data of related compounds and confirmed by the mass peak and its isotope composition in GCMS. When an excess of reagents were used the starting compound was completely consumed and both chlorinated products were formed in a ratio **10**:**11** of 47:53 (Entry 4). As TFE is a polar solvent the decomposition of (dichloroiodo)arene **9b** to the ring-chlorinated products **10** or **11** during further stirring of the reaction mixture was not surprising. However, an electrophilic attack by the positive chlorine species on the aromatic nucleus cannot be excluded. In the absence of TFE, H_2_O_2_ was not able to oxidize HCl and after 20 h the starting compound **9a** was recovered. Similar results were obtained in reaction with molecular chlorine in hexane, where reaction took place at the iodine atom to yield **9b**, which was isolated by filtration in 45% yield, but is not stable in solution and it decomposes to form a mixture of chlorinated products. 

**Table 2:** Chlorination of 2,4-dimethyliodobenzene (**9a**).

			Distribution^b^ (%)
Entry	Method^a^	Time (h)	9a : 9b^c^ : 10 : 11
1	A	0.5	83 : 17 : / : /
2	A	2	54 : 46 : trace : trace
3	A	20	33 : / : 30 : 37
4	B	20	/ : / : 47 : 53
5	D	6	19 : 22 : 28 : 31

Reaction conditions: **9a** (1.0 mmol), ambient temperature. ^a ^Method A: conc. HCl (2.0 mmol), 30% aq. H_2_O_2_ (1.0 mmol), TFE (1.0 mL); Method B: conc. HCl (4.0 mmol), 30% aq. H_2_O_2_ (2.0 mmol), TFE (1.0 mL); Method C: conc. HCl (4.0 mmol), 30% aq. H_2_O_2_ (4.0 mmol), TFE (1.0 mL). Method D: Cl_2_, hexane (10 mL). ^b ^Distribution of products determined by ^1^H-NMR spectroscopy. ^c ^**9b** was determined by its characteristic signals for ArICl_2_ compounds in ^1^H-NMR spectra of the reaction mixture – 8.07 ppm (d, *J* = 8 Hz, 1H).

**Scheme 3:**
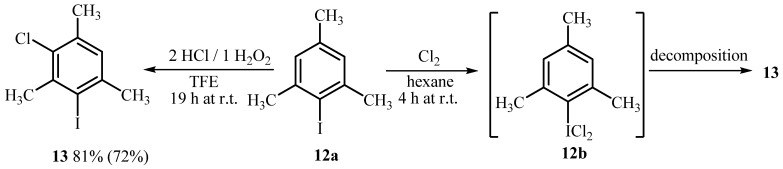
Chlorination of 2,4,6-trimethyliodobenzene (**12a**).

Furthermore, more activated 2,4,6-trimethyliodobenzene (**12a**) was studied (Scheme 3). Contrary to **9a**, when **12a **reacts with 2.0 mol equiv. of HCl and 1.0 mol equiv. of H_2_O_2_ in TFE, **12b** is not detected in the ^1^H-NMR spectra of the reaction mixture and a new signal was observed at 6.99 ppm which was attributed to 1-chloro-5-iodo-2,4,6-trimethylbenzene (**13**) (16% and 29% yield after 0.5 h and 2 h, respectively). When oxidative chlorination was performed for 19 h 81% of **13** was formed and isolated in 72% yield (Scheme 3). The structure of **13** was determined by comparison with the literature data of structurally related data and GCMS analysis (mass signal and its isotope distribution). Very similar result was observed on chlorination of **12a** with Cl_2_ in hexane and **12b** could be isolated only after the reaction was performed in CHCl_3_, as already reported [36]. On standing at room temperature, **12b** was completely converted in the product **13**. We can conclude from this that **12b** is insufficiently stable to be detected by NMR and it most likely decomposes with concomitant ring chlorination into **13**.

**Scheme 4:**
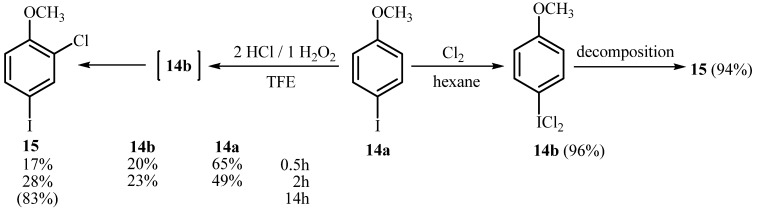
Chlorination of 4-iodoanisole (**14a**).

According to these results, the formation of an electrophilic substitution product in oxidative chlorination of iodoarenes containing a strong electron-donating group, like a methoxy moiety, in 1,1,1-trifluoroethanol, is not surprising. 4-Methoxyiodobenzene (**14a**) is converted with 2.0 mol equiv. of H_2_O_2_ and 1.0 equiv. of HCl in 14 h to chlorinated product **15**, isolated in 83% yield, most likely *via* the formation of (dichloroiodo)arene **14b** as an intermediate product. Interestingly, the product **14b** was detected by ^1^H-NMR of the reaction mixture, where it was identified by a characteristic signal for ArICl_2_ compounds, namely 8.06 ppm (d, *J* = 9 Hz) and 6.95 ppm (d, *J* = 9 Hz). However the direct chlorination of **14a** in polar TFE to give the chlorinated product **15 **cannot be excluded. In contrast, in chlorination with chlorine only iodine dichloride **14b** in a 96% yield was isolated (Scheme 4). Nevertheless, **14b** is unstable and ^1^H-NMR analysis reveals clearly its rapid decomposition with concomitant ring chlorination. Interestingly, when using other oxidants like SPB [21], UHP [22], KClO_3_ [17] or Na_2_S_2_O_3_ [18] in combination with HCl, (dichloroiodo)-4-metoxybenzene (**14b)** is obtained in good yields, and there is no observable chlorination at the aromatic nucleus. In some cases CCl_4_ was used rather than polar solvents [18,21]. However, it must be emphasized that it is preferable to obtain (dichlororiodo) arenes from iodoanisole and other highly activated iodoarenes by either using biphasic conditions or the classic Willgerodt method, where ArI and molecular chlorine are both dissolved and react in an inert solvent [18]. Alternatively, appropriate liquid phase oxidative chlorination protocols seem to be more suitable for deactivated aryliodides, since such substrates need to be activated by the solvent effect of TFE in order to react.

### 2.3. Investigation of the Nature of Reactive Chlorinating Species

**Scheme 5:**
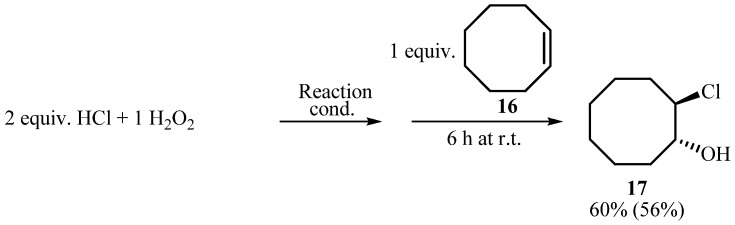
Oxidative chlorination of cyclooctene (**16**).

An investigation was then made into the transformation of aryl iodides into aryliodine(III) dichlorides by an oxidative chlorination pathway. As already mentioned, when aryliodine dichlorides were synthesized using a combination of an urea hydrogen peroxide complex and hydrochloric acid in glacial acetic acid, the authors anticipated that chlorine is formed *in situ* [16]. In a similar reaction under solvent-free conditions at 85 °C the same authors suggested that the composite mixtures of urea with iodosylarenes, ArIO, and probably also admixed with iodylarenes, ArIO_2_ is formed [22]. After cooling, the resultant melts were added portion-wise to excess concentrated hydrochloric acid, which resulted in the formation of the desired (dichloroiodo)arenes. In our case, after the addition of concentrated HCl to a suspension of aryl iodide and H_2_O_2_ in TFE a yellow color appeared indicating the oxidation of HCl took place. The occurrence of this initial stage of reaction was confirmed by addition of cyclooctene (**16**) to HCl/H_2_O_2_/TFE system where it was transformed into the corresponding chlorohydrin **17** (Scheme 5, Table 3, entry 1). When the same chlorination was performed without TFE, reaction mixture had to be heated to initiate the transformation (Table 3, entry 2). Therefore, we can conclude that H_2_O_2_ is activated to the same extent either with TFE or by heating. For comparison, reactions using UHP as an oxidant were performed. At room temperature only 5% of **17** was formed (Entry 3), while heating to 85 °C increased conversion only to 17% (Entry 4). This result was curious because similar reaction conditions were used in solvent-free synthesis of PhICl_2_ [22]. One possibility is that UHP is activated by heating at 85 °C for 30 min prior to the reaction. When thus activated UHP was used for oxidative chlorination of **16** reaction proceeded (Entry 5). This might indicate that small changes in the structure of the UHP complex effects its activity. Again, by using TFE no additional activation is necessary and oxidative chlorination of **16** with UHP and HCl occurs at room temperature (Entry 6).

**Table 3:** Oxidative chlorination of cyclooctene (**16**).

Entry	Oxidant	React. cond.^a^	Conv.^b^ (%)
1	H_2_O_2_	TFE^c^, 2 h at r.t.	60
2	H_2_O_2_	2 h at 85°C	62
3	UHP	2 h at r.t.	5
4	UHP	2 h at 85°C	17
5	UHP^d^	2 h at r.t.	38
6	UHP	TFE^c^, 2 h at r.t.	84

^a^ Reaction conditions: 2.0 mmol (0.164 mL) of conc. HCl was added to 1.0 mmol (0.102 mL) of 30% aq. H_2_O_2_ or 1.0 mmol (94 mg) UHP, after quoted time 1.0 mmol (110 mg) of cyclooctene (**16**) was added and stirred for 2 h at room temperature. ^b^ Conversion of **16** by ^1^H-NMR spectroscopy. ^c^ 1 mL of TFE. ^d^ 1.0 mmol (94 mg) of powdered UHP was heated for 30 min at 85 °C, after cooling 2.0 mmol (0.164 mL) of conc. HCl and 1.0 mmol (110 mg) of **16** were added.

**Scheme 6:**
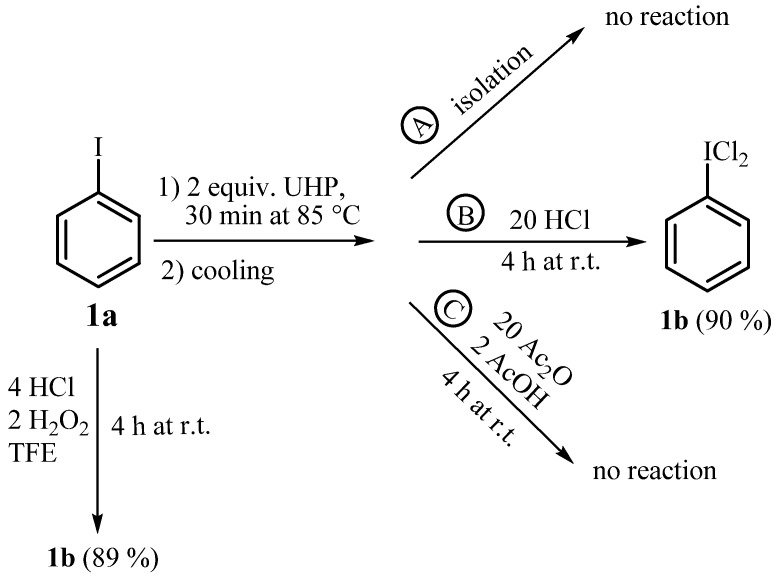
Oxidative transformations of iodobenzene (**1a**).

The above results point to the conclusion that TFE activates hydrogen peroxide for oxidation of HCl at ambient temperature, regardless of the form of peroxide. Next, we approached the study from a different point and investigated the possibility of oxidation of iodobenzene (**1a**) with hydrogen peroxide and UHP complex as a first step of the reaction. In the solvent-free reaction synthesis of (dichloroiodo)arenes with UHP/HCl it was proposed that the first step of transformation is oxidation of iodoarene [22]. However, it is known that the oxidation of the iodine atom of aryliodide is difficult to achieve and harsh reaction conditions are usually required [1]. In the reaction between **1a** and 2 equiv. of UHP at 85 °C for 30 min only the starting compound was recovered after isolation (Scheme 6, Path A). Also, no reaction occurred when **1a** was stirred with 2 equiv. of H_2_O_2_ in TFE. Nevertheless, when concentrated HCl was added to the preheated mixture from the example A on Scheme 6, 90% of PhICl_2_ was isolated (Path B). However, when HCl was replaced by acetic anhydride in combination with acetic acid (Path C) no reaction occurred. This is further proof that PhIO is not formed, since it is known that PhIO after treatment with AcOH produces (diacetoxyiodo)benzene [32].

## 3. Experimental Section

All chemicals were obtained from commercial sources and used without further purification. Column chromatography was carried out using Silica gel 60 (0.063–0.200 mm). ^1^H- and ^13^C-NMR spectra were recorded in CDCl_3_ using a Varian Inova 300 MHz spectrometer operating at 300 and 76 MHz, respectively.. The chemical shifts (δ) are reported in ppm units relative to TMS as an internal standard for ^1^H-NMR and CDCl_3 _for ^13^C-NMR spectra. Melting points were determined using a Büchi 535 melting point apparatus. GCMS were performed with GC - HP 6890: capilary column DB-5 MS length 30 m, ID 0,25 mm, 0,25 μm (5% fenil-methylpolysyloxane) stacionary phase, detection: EI (70eV).

### 3.1. General Reaction Procedure for the Synthesis of Aryliodine(III) Dichlorides **1b-8b** with HCl H_2_O_2_ (Methods A, B and C, Table 1)

In a typical experiment aryliodide **1a-8a** (1.0 mmol) was suspended in 1,1,1-trifluoroethanol (1 mL) to which 30% aqueous H_2_O_2_ (0.102 mL, 1.0 mol equiv., Method A or 0.204 mL, 2.0 mol equiv., Method B, or 0.408 mL, 4.0 mol equiv., Method C) and 37% aqueous hydrochloric acid (0.164 mL, 2.0 mol equiv., Method A, or 0.328 mL, 4.0 mol equiv., Methods B and C) were added. The mixture was stirred at room temperature for 4-24 h. The progress of the reaction was followed by TLC. At the end of the reaction 1,1,1-trifluoroethanol was removed under reduced pressure to give a yellowish precipitate, which was filtered off and washed with 5 mL of distilled water. The product was then air-dried, analyzed by ^1^H- and ^13^C-NMR spectroscopy and its melting point measured. The purity of the aryliodine(III) dichlorides was determined by iodometric titration, which afforded the starting aryl iodides as determined by ^1^H-NMR spectroscopy.

### 3.2. General Reaction Procedure for the Synthesis of Aryliodine(III) Dichlorides **1b-8b** with Cl2 in Hexane (Method D, Table 1)

In a typical experiment aryl iodide **1a-8a** (1.0 mmol) was dissolved in hexane (10 mL). The solution was saturated with molecular chlorine and then stirred at room temperature for 3–9 h under an atmosphere of excess chlorine. The precipitate was filtered off, air-dried and analyzed by ^1^H-NMR.

*(Dichloroiodo)benzene *(**1b**): Yellow solid; yields: Method A: 220 mg (80%), Method B: 245 mg (89%), Method C: 264 mg (96%); mp (capillary) 118–120 °C (110–112 °C [23]); ^1^H-NMR (CDCl_3_) 7.44–7.51 (m, 2H, Ar-H), 7.57–7.63 (m, 1H, Ar-H), 8.17–8.21 (m, 2H, Ar-H); ^13^C-NMR (CDCl_3_) 122.8, 131.6, 132.1, 133.8. Purity 99%.

*4-Methyl(dichloroiodo)benzene *(**2b**): Yellow solid; yields: Method A: 191 mg (66%), Method B: 260 mg (90%), Method D: 266 mg (92%); mp (capillary): 110–112 °C (108–118 °C [22]); ^1^H-NMR (CDCl_3_) 2.46 (s, 3H, CH_3_), 7.26 (d, *J* = 8.6 Hz, 2H, Ar-H), 8.04 (d, *J* = 8.6 Hz, 2H, Ar-H); ^13^C-NMR (CDCl_3_) 21.4, 121.9, 132.3, 133.8, 137.2. Purity 97%.

*4-tert-Butyl(dichloroiodo)benzene *(**3b**): Yellow solid; yields: Method A: 182 mg (55%), Method C: 265 mg (80%), Method D: 281 mg (85%); mp (capillary): 88–90 °C (84 °C [33]); ^1^H-NMR (CDCl_3_) 1.34 (s, 9H, C(CH_3_)_3_), 7.47 (d, *J* = 8.8 Hz, 2H, Ar-H), 8.08 (d, *J *= 8.8 Hz, 2H, Ar-H); ^13^C-NMR (CDCl_3_) 31.1, 35.2, 121.8, 127.5, 128.9, 133.6. Purity 95%.

*4,4’-bis(Dichloroiodo)biphenyl *(**4b**): Yellow solid; yields: Method B: 464 mg (85%), Method C: 486 mg (89%), Method D: 524 mg (96%); mp (capillary): 153 °C (153 °C [7]). Purity 98%.

*3-(Dichloroiodo)benzoic acid *(**5b**): Yellow solid; yields: Method A: 128 mg (40%), Method B: 249 mg (78%), Method D: 262 mg (82%); mp (capillary): 187–190 °C (187–190 °C [7]); ^1^H-NMR (CDCl_3_) 7.33–7.38 (m, 1H), 8.00–8.08 (m, 2H), 8.30–8.34 (m, 1H); ^13^C-NMR (CDCl_3_) 129.8, 131.5, 133.8, 134.4, 139.23, 142.7, 166.1. Purity 98%.

*3-Nitro(dichloroiodo)benzene* (**6a**): Yellow solid; yields: Method A: 64 mg (20%), Method B: 230 mg (72%), Method D: 242 mg (76%); mp (capillary): 91–94 °C (90 °C [21]); ^1^H-NMR (CDCl_3_) 7.72 (dd, *J* = 8.3, 8.3 Hz, 1H, Ar-H), 8.46 (ddd, *J* = 8.3, 2.0, 0.9 Hz, 1H, Ar-H), 8.55 (ddd, *J* = 8.3, 2.0, 0.9 Hz, 1H, Ar-H), 9.09 (dd, *J* = 2.0, 2.0 Hz, 1H, Ar-H); ^13^C-NMR (CDCl_3_) 123.2, 126.8, 129.0, 132.0, 139.0. Purity 100%.

*3-Chloro(dichloroiodo)benzene* (**7a**): Yellow solid; yields: Method A: 183 mg (59%), Method C: 263 mg (85%), Method D: 279 mg (90%); mp (capillary): 95–96 °C (92–94 °C [34]); ^1^H-NMR (CDCl_3_) 7.42 (dd, *J* = 8.2, 8.2 Hz, 1H, Ar-H), 7.57 (ddd, *J* = 8.2, 1.9, 0.8 Hz, 1H, Ar-H), 8.10 (ddd, *J* = 8.2, 1.9, 0.8 Hz, 1H, Ar-H), 8.19 (dd, *J* = 1.9, 1.9 Hz, 1H, Ar-H); ^13^C-NMR (CDCl_3_) 124.0, 131.7, 132.1, 132.6, 133.5, 136.4. Purity 100%.

*4-Chloro(dichloroiodo)benzene *(**8a**): Yellow solid; yield; Method A: 155 mg (50%), Method C: 263 mg (85%), Method D: 285 mg (92%); mp (capillary): 108–110 °C (110–112 °C [23]); ^1^H-NMR (300 MHz; CDCl_3_) 7.44 (d, *J* = 8.9 Hz, 2H, Ar-H), 8.11 (d, *J* = 8.9 Hz, 2H, Ar-H); ^13^C NMR (76 MHz; CDCl_3_) 121.8, 131.7, 135.1, 138.9. Purity 96%.

*2,4-Dimethyl(dichloroiodo)benzene *(**9b**): Yellow unstable solid; yields: Method D: 136 mg (45%); mp (capillary): 94–96 °C (95–98.5 °C [23]). NMR could only be obtained for the reaction mixture and signals for **9b** were assigned by comparison with known analogous compounds: ^1^H-NMR (CDCl_3_) 2.43 (s, 3H), 2.80 (s, 3H), 7.06 (m, 1H), 7.29 (m, 1H), 8.07 (d, *J* = 8.2 Hz, 1H). Compound **9b** decomposes in the solution and only a mixture of two chlorinated products, **10** and **11**, can be isolated by preparative TLC (SiO_2_, hexane) from the reaction mixture obtained after 20 h reaction under method A in a yield of 35% (93 mg). Products were determined by comparison of the ^1^H-NMR shifts with the literature data of related compounds and confirmed by GCMS (intensity of peaks M:M+2 = 3:1) [35].

*1-Chloro-5-iodo-2,4-dimethylbenzene* (**10**) ^1^H-NMR (CDCl_3_) 2.23 (s, 3H), 2.37 (s, 3H), 7.06 (s, 1H) and 7.79 (s, 1H), and *2-Chloro-4-iodo-1,3-dimethylbenzene* (**11**) ^1^H-NMR (CDCl_3_) 2.33 (s, 3H), 2.60 (s, 3H), 6.78 (d, *J* = 8.2 Hz, 1H), 7.60 (d, *J* = 8.2 Hz, 1H); GCMS (EI) 266 (M, 100), 268 (M+2, 35), 231 (10), 141 (19), 139 (58), 103 (52), 77 (42).

*2,4,6-Trimethyl(dichloroiodo)benzene *(**12b**) [36]: Yellow unstable solid; yield: Method D (in CHCl_3_): 63 mg (20%); mp (capillary): 67–68 °C. After standing for 24 h **12b** decomposed and only one product was formed. It was identical to the product obtained by Method A and characterized by comparison of the NMR shifts with the literature data of related compounds and by GCMS (intensity of peaks M:M+2 = 3:1) as *1-chloro-5-iodo-2,4,6-trimethylbenzene* (**13**) [37]: Yield: Method A, 274 mg (72%); ^1^H-NMR (CDCl_3_) 2.28 (s, 3H), 2.39 (s, 3H), 2.62 (s, 3H), 6.99 (s, 1H). ^13^C-NMR (CDCl_3_) 20.7, 27.7, 29.7, 105.3, 127.9, 129.0, 135.8, 138.9, 140.0; GCMS (EI) 280 (M, 100), 282 (M+2, 42), 245 (13), 155 (14), 153 (44), 115 (46), 91 (25).

*4-Methoxy(dichloroiodo)benzene *(**14b**): Yellow unstable solid; yield: Method D: 293 mg (96%); mp (capillary): 58–61 °C (75–78 °C [23]). NMR could only be obtained for the reaction mixture and signals for **14b** were elucidated by a comparison with known analogous compounds: ^1^H-NMR (CDCl_3_) 3.87 (s, 3H), 6.94 (d, *J* = 9 Hz, 2H), 8.05 (d, *J* = 9.0 Hz, 2H). **14b** decomposes in the solution into a chlorinated product **15**. 

*2-Chloro-4-iodoanisole *(**15**) [38]: Yield; Method A: 223 mg (83%). ^1^H-NMR (CDCl_3_) 3.88 (s, 3H, OCH_3_), 6.68 (d, *J* = 8.6 Hz, 1H, Ar-H), 7.51 (dd, *J* = 8.6, 2.2 Hz, 1H, Ar-H), 7.66 (d, *J* = 2.2 Hz, 1H, Ar-H).

### 3.3. Oxidative chlorination of cyclooctene (**16**, Scheme 6)

Aqueous HCl (37 %, 0.164 mL, 2.0 mmol) and either 30% aq. H_2_O_2_ (0.204 mL, 2.0 mol) or urea hydrogen peroxide (188 mg, 2.0 mmol) were mixed together according to the conditions listed underneath the Table 2. Then cyclooctene (**16**, 110 mg, 1.0 mmol) was added and the reaction mixture was further stirred for 6 h at ambient temperature. The reaction mixture was isolated by extraction with CH_2_Cl_2_ and analyzed by ^1^H-NMR spectroscopy.

*2-Chlorocyclooctanol *(**17**) [39]: From **16 **(1.0 mmol), H_2_O_2_ (2. 0 mmol), HCl (2.0 mmol) and 1,1,1-trifluoroethanol (1 mL). The product was isolated by column chromatography (SiO_2_, CH_2_Cl_2_). Yield: 90 mg (56%). ^1^H-NMR (MHz; CDCl_3_) 1.30–2.30 (m, 12H), 3.87 (m, 1H), 4.10 (ddd, *J* = 9.3, 7.2, 2.7 Hz, 1H).

### 3.4. Oxidative transformations of iodobenzene (**1a**, Scheme 6)

Iodobenzene (**1a**, 204 mg, 1.0 mmol) was added to the finely powdered urea hydrogen peroxide (188 mg, 2.0 mmol) and the mixture was stirred for 30 min at 85 °C. After cooling to room temperature the procedure quoted in Scheme 6 followed; Path A: Isolation of the reaction mixture by extraction with CH_2_Cl_2_, Path B: Addition of 37% aq. HCl (1.64 mL, 20.0 mmol) and stirring at room temperature for 4 h. The yellow precipitate was then washed with water. Path C: Addition of Ac_2_O (1.89 mL, 20.0 mmol) and AcOH (0.114 mL, 2.0 mmol), stirring at room temperature for 4 h. In all cases the isolated reaction mixture was analyzed by ^1^H-NMR spectroscopy and the product determined by comparison of chemical shifts with literature.

## 4. Conclusions

Reported herein is a simple method for the synthesis of aryliodine(III) dichlorides from various aryl iodides under mild reaction conditions, in which a combination of aqueous hydrochloric acid and aqueous hydrogen peroxide in 1,1,1-trifluoroethanol was used. The use of H_2_O_2_ as a ‘‘green’’ oxidant produces water as the only by-product, which not only increases the overall atom economy but also facilitates subsequent isolation of the product. Furthermore, 1,1,1-trifluoroethanol as a reaction medium and a template catalyst activates H_2_O_2_ for the oxidation of HCl and enables reactions to occur at room temperature. It has also been established that, in this system, chlorine is formed initially that then reacts with aryl iodide to give aryliodine(III) dichloride in yields comparable with those obtained by other oxidative methods using either stronger oxidants or molecular chlorine. This, together with the already mentioned advantages makes it a good alternative to existing methods. From a study into what effect the substituents on the aromatic ring have on yield we can conclude that the presence of electron-withdrawing groups result in a slower reaction and lower yields of (dichloroiodo)arenes. On the contrary, in the case of chlorination of iodoarenes with electron-donating substituents (*i.e*., methoxy), ring chlorinated products were formed together with (dichloroiodo)arenes, most likely from their decomposition in polar 1,1,1-trifluoroethanol. 
